# Impact of control selection strategies on GWAS results: a study of prostate cancer in the UK biobank

**DOI:** 10.1093/bib/bbag266

**Published:** 2026-05-31

**Authors:** Jingzhan Lu, Johan H Thygesen, Robin N Beaumont, Michael N Weedon, Harry D Green

**Affiliations:** Department of Clinical and Biomedical Sciences, University of Exeter, 79 Heavitree Road, Exeter, EX1 2LU, United Kingdom; Institute of Health Informatics, University College London, 210 Euston Road, London, NW1 2DA, United Kingdom; Institute of Health Informatics, University College London, 210 Euston Road, London, NW1 2DA, United Kingdom; Department of Clinical and Biomedical Sciences, University of Exeter, 79 Heavitree Road, Exeter, EX1 2LU, United Kingdom; Department of Clinical and Biomedical Sciences, University of Exeter, 79 Heavitree Road, Exeter, EX1 2LU, United Kingdom; Department of Clinical and Biomedical Sciences, University of Exeter, 79 Heavitree Road, Exeter, EX1 2LU, United Kingdom

**Keywords:** GWAS, control selection, matching analysis, biobank, methodology, prostate cancer

We thank Law et al. for their insightful correspondence regarding our article, ‘Impact of control selection strategies on GWAS results: a study of prostate cancer in the UK Biobank’. We appreciate the opportunity to engage in this scholarly dialogue, as their comments highlight critical theoretical considerations in the evolving landscape of large-scale genomic studies.

While we recognize the concerns raised regarding the historical use of propensity score matching (PSM) in causal inference, we respectfully suggest that matching is also fundamentally a sampling and design-stage strategy. In the context of epidemiological study design, matching does not, by itself, define a causal estimand nor impose a causal interpretation. Its appropriateness should be determined by the inferential target and empirical performance within a specific study framework.

As noted in established epidemiological principles (e.g. *BMJ 2005;330:1018*), matching in case–control studies is primarily employed to enhance study efficiency and precision [1]. By ensuring that cases and controls are balanced across key strata such as age, sex, or recruitment timing, matching ensures that the distribution of confounders is roughly equal and thereby maximizes the statistical power of the comparison within those strata. Furthermore, matching can be a pragmatic tool to control for unquantifiable or complex factors (e.g., granular socioeconomic position or technical batch effects) that are difficult to parameterize in a standard regression model. Therefore, rather than being a tool exclusive to causal inference, we view PSM as a sophisticated extension of traditional case–control matching, designed to achieve balanced groups in high-dimensional data settings like the UK Biobank.

Regarding the concern of collider bias from genetic Principal Components (PCs), we acknowledge that over-conditioning can theoretically attenuate or enhance signals. However, incorporating PCs is standard practice in GWAS to control for population stratification [2]. Empirical research highlights the risk of ‘index event bias’ (a form of collider bias) in large biobanks; e.g. in 113,203 non-diabetic UK Biobank participants, type 2 diabetes risk alleles (e.g. near TCF7L2, CDKN2AB, and CDKAL1) showed spurious or overestimated associations with decreased BMI [3]. This demonstrates that stratifying by or enriching for disease status can lead to false-positive genetic associations that even reach genome-wide significance in large-scale studies. We do not argue that matching is a theoretical necessity for unbiased estimation; rather, we evaluated it as a design-stage choice to balance the cost and GWAS power.

It is critical to note that conditioning via regression adjustment and via sample selection are not equivalent. The actual impact of collider bias is an empirical question, not a conceptual one. In large-scale biobanks with extreme case–control imbalance, relying solely on standard estimators can lead to numerical instability or the failure of asymptotic approximations. In such settings, matching can complement mixed-model approaches by ensuring a more balanced and stable comparison group, thereby enhancing statistical robustness.

Our analyses explicitly evaluated the impact of matching on downstream association results, rather than assuming either benefit or harm a priori. In theory, we agree that aggressive matching could reduce power for rare-variant analyses if it substantially restricts the sample or conditions on fine-scale ancestry. However, our manuscript did not advocate matching as a general strategy for rare-variant discovery but rather assessed its performance under specific analytical settings. For researchers seeking a cost-effective and computationally efficient method to replicate established GWAS hits, the practical utility of matching remains significant; this loss is often reasonable for the replication of large-effect GWAS signals.

In conclusion, we believe the key question is not whether matching ‘belongs’ to GWAS in principle, but rather under which circumstances specific control selection strategies improve or degrade empirical performance. We value the perspective provided by Law *et al.* and agree that a cautious approach is necessary when applying design-stage conditioning. However, as the field increasingly shifts toward more complex genomic studies involving multiple cohorts and linked to electronic health records (EHRs), it remains important to explore the utility of paired analysis as a cornerstone of classical epidemiology. Strategic sample selection offers a pragmatic balance between cost efficiency and analytical depth in an era of increasingly massive data.

Yours sincerely,

Jingzhan Lu and Harry Green (on behalf of all co-authors).

**Key Points**

- Propensity score matching (PSM) serves as a robust sampling and design-stage strategy to enhance study efficiency and handle covariate balance in biobank-scale data.
- While conditioning on genetic components can theoretically risk collider bias, the practical impact is an empirical trade-off between mitigating population stratification and preserving statistical signal.
- Matching offers a computationally efficient and cost-effective approach for replicating Genome-Wide Association Study (GWAS) signals, particularly in settings of extreme case–control imbalance.
